# Confronting the Neglected Problem of Snake Bite Envenoming: The Need for a Global Partnership

**DOI:** 10.1371/journal.pmed.0030150

**Published:** 2006-06-06

**Authors:** José María Gutiérrez, R. David G Theakston, David A Warrell

## Abstract

Envenoming resulting from snake bites is an important public health hazard in many regions of the world, yet public health authorities have given little attention to the problem.

Envenoming resulting from snake bites is an important public health hazard in many regions, particularly in tropical and subtropical countries [ 1–3]. Although antivenoms are being produced by various laboratories in every continent, the burden of snake bite envenoming—causing both morbidity and mortality—still has a great impact on the population and on health-care systems, especially in Africa, Asia, Oceania, and Latin America [ 4]. Unfortunately, public health authorities, nationally and internationally, have given little attention to this problem, relegating snake bite envenoming to the category of a major neglected disease of the 21st century.

## The Global Burden of Snake Bite Envenoming

In the tropical developing countries where snake bites occur most commonly, there are few reliable incidence data. One serious attempt to assess global snake bite mortality was the survey undertaken by Swaroop and Grabb in 1954, which was based largely on hospital admissions [ 3]. Subsequent work has revealed gross underreporting of deaths in this study—for example, in Nigeria [ 5] and Thailand [ 1, 6]. One reason is that records of patients treated by traditional methods are missing from official databased statistics, and deaths reported at the hamlet or district level may not be sent on to ministry headquarters. Accepting these limitations, the fragmentary evidence available suggests that several million bites and envenomings occur worldwide each year, with tens of thousands of deaths [ 2, 3].

Properly designed population surveys are likely to give a far more accurate picture of snake bite incidence, morbidity, and mortality. In Asia, the highest recorded mortality was 162 snake bite deaths per 100,000 people per year, in the Eastern Terai of Nepal [ 7]. In Africa, for instance, the incidence of snake bites in the Benue Valley of northeastern Nigeria was 497 per 100,000 people per year, with a mortality of 12.2% [ 5]. The reasons for the high levels of snake bite mortality in tropical developing countries include scarcity of antivenoms, poor health services, and difficulties with rapid access to health centres [ 8]. Large numbers of victims survive with permanent physical and psychological sequelae, mostly due to the tissue-damaging effects of snake venoms. Young agricultural workers, especially males, are the most highly affected group, making snake bite envenoming a truly occupational disease—a fact that is frequently overlooked by national authorities. Children are also common victims of snake bites. There is significant seasonal variation in snake bite incidence that is attributable to climate, especially to rain fall and temperature, which determine annual cycles of agricultural activity.

## Species That Cause High Morbidity and Mortality

**Figure 1:**
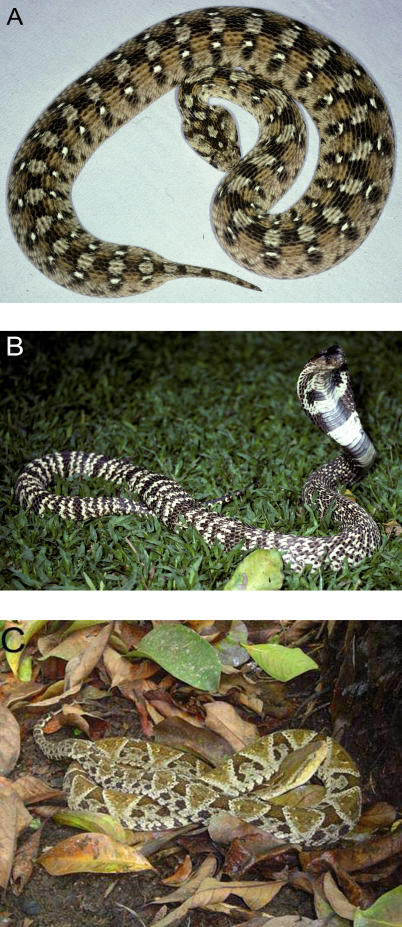
Snakes Causing High Incidence of Envenomings in Africa, Asia, and Latin America (A) Echis ocellatus (saw-scaled viper, family Viperidae, from Nigeria), (B) Naja naja (cobra, family Elapidae, from Sri Lanka), and (C) B. asper (terciopelo or equis, family Viperidae, from Costa Rica) are responsible for many snake bite envenomings in sub-Saharan Africa, Asia, and Central America, respectively. Envenomings by E. ocellatus and B. asper, as well as by related viperid species, induce prominent local tissue damage that often results in permanent sequelae and disability, and systemic alterations associated with haemorrhage, coagulopathy, cardiovascular shock, and renal failure. Envenomings by N. naja and other Asiatic cobras induce systemic neurotoxic effects and local necrosis. (Image: D. A. Warrell [A and B]; Mahmood Sasa [C])

Most severe cases of snake bite envenoming are inflicted by species of the family Elapidae (cobras, kraits, mambas, Australasian species, and sea snakes) and the family Viperidae (rattlesnakes, lance-headed pit vipers, and true vipers). The species causing the largest numbers of bites and fatalities are Echis sp. (saw-scaled vipers) in northern Africa, Bothrops asper and B. atrox (lance-headed pit vipers) in Central and South America, and Naja sp. (cobras) and Bungarus sp. (kraits) in Asia [ 6, 9–11] ( Figure 1). Many other species, although not responsible for a large number of cases worldwide, constitute a serious problem in specific regions.

## Clinical Features of Snake Bite Envenoming

**Figure 2:**
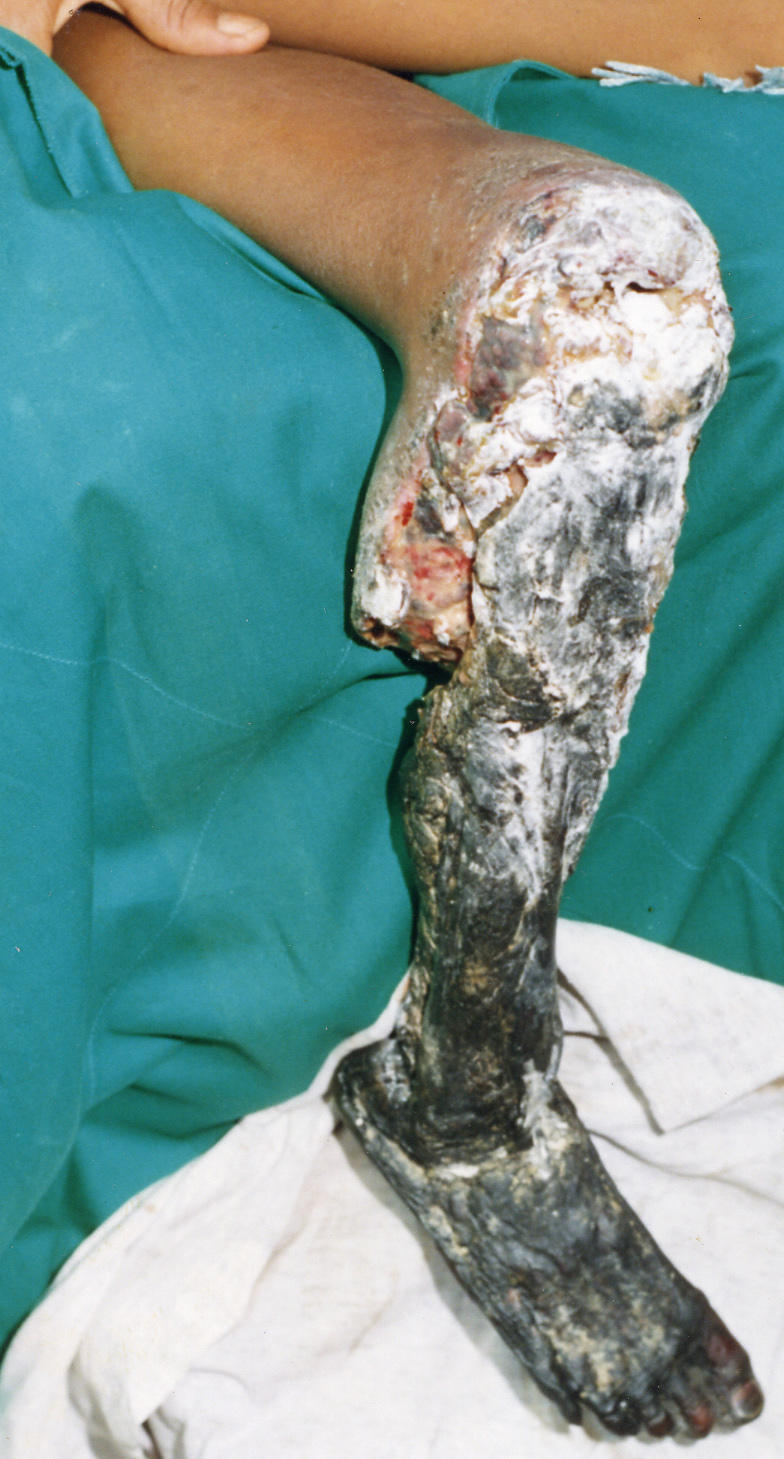
Extensive Tissue Necrosis of the Lower Limb in an 11-Year-Old Boy Who Had Been Bitten Two Weeks Earlier by a B. asper in Ecuador Only antibiotic treatment had been given, so tissue damage was extensive, requiring above-knee amputation. Such pathological alterations are caused by the direct action of locally acting toxins in the venom (mostly metalloproteinases and phospholipases A _2_), together with an indirect effect promoted by local inflammatory events and ischemia secondary to venom-induced vascular damage and increased intracompartmental pressure. (Image: D. A. Warrell)

Envenomings by most viperid snakes inflict prominent local tissue damage, characterised by swelling, blistering, haemorrhage, and necrosis of skeletal muscle ( Figure 2). Bites by some elapids, such as African spitting cobras and some Asian cobras, also induce local necrosis. Such local pathology is mostly due to the action of phospholipases A _2_ and zinc-dependent metalloproteinases [ 12, 13]. These local effects develop rapidly after the bite; consequently, a delay in the access to health facilities frequently results in drastic tissue damage and permanent disability. Systemic effects of envenoming are also common and more immediately life-threatening. In elapid snake bites, neurotoxicity is a typical consequence, attributable to effects of pre- and/or postsynaptically acting neurotoxins targeting neuromuscular junctions. These toxins cause progressive descending paralysis, which may become life-threatening when bulbar and respiratory muscles are involved. In envenomings by several elapid snakes and some viperids, such as South American rattlesnakes, there is a generalised rhabdomyolysis induced by phospholipases A _2_, which may cause myoglobinaemia, hyperkalaemia, and acute renal failure. Systemic effects in viperid snake bites include spontaneous haemorrhage—cerebral haemorrhage being the most serious manifestation—defibrinogenation, disseminated intravascular coagulation, and cardiovascular shock secondary to hypovolaemia, vasodilation, and direct effects on the myocardium. Acute renal failure and acute respiratory distress syndrome are also described following viperid snake bites. There are abundant exceptions to this general pathophysiological profile since the amazing complexity of snake venom proteomes includes toxins with diverse clinical effects (see [ 14] for a general view on the clinical manifestations of snake bite envenomings).

## Antivenoms and Ancillary Treatments in Envenoming

The intravenous administration of animal-derived (mostly horse or sheep) antivenoms is the mainstay and the only specific treatment of snake bite envenoming. Many laboratories produce antivenoms using various methodologies, which yield three different types of products depending on the nature of the active principle. In some cases, antivenoms consist of whole IgG molecules, but the majority of producers manufacture antivenoms composed of F(ab') _2_ fragments obtained by pepsin digestion of IgG, and a few laboratories produce Fab fragments after papain digestion of IgG [ 4, 15]. Variations in the molecular mass of the active principle, as well as other immunological divergences among these types of antivenoms, determine different pharmacokinetic profiles, with evident pharmacodynamic implications—such as the described recurrence of envenoming—especially when using Fab antivenoms [ 16]. The large immunochemical diversity of snake venoms explains why the efficacy of antivenoms is generally restricted to a limited geographical and biological spectrum.

Clinical investigations have established that generally antivenoms are highly effective in the neutralisation of toxins responsible for systemic effects such as haemorrhage, coagulopathy, and haemodynamic disturbances. Similarly, a rapid reversal of neurotoxicity caused by postsynaptic neurotoxins in elapid envenomings is well established [ 1]. In contrast, antivenoms are not very effective in the reversal of neurotoxicity induced by presynaptically acting phospholipases A _2_. The main problem associated with antivenom efficacy has to do with local tissue damage [ 1]. This problem is not due to the lack of neutralizing antibodies in antivenoms, but rather to the extremely rapid development of local pathology, which renders it difficult for neutralizing antibodies to access the area before irreversible damage occurs [ 17].

Antivenom safety is also of current concern since, although the safety profile of some products is satisfactory, the administration of other antivenoms is associated with a high incidence of early and late adverse reactions [ 4, 15]. The precise mechanisms responsible for these reactions have not been clearly established, although there is strong evidence pointing to direct complement activation, effects of contaminating pyrogens, and reactions to immune complexes. The physicochemical characteristics of antivenoms—principally, the presence of protein aggregates, the purity of the active principle, and the total dose of protein administered—seem to be the main determinants of reactogenicity. Early adverse reactions are treated with adrenaline, antihistamines, or corticosteroids [ 6, 9].

Besides antivenom administration, the therapy of snake bite envenoming involves a number of ancillary interventions [ 6]. Assisted ventilation is life saving when the respiratory muscles are affected, and administration of acetylcholinesterase inhibitors has been used with success in some neurotoxic envenomings [ 6]. Hypovolaemia in viperid envenoming is corrected by the administration of plasma expanders, and dialysis or haemoperfusion may be needed in cases of acute renal failure. Renal damage by myoglobin or haemoglobin released by rhabdomyolysis or intravascular haemolysis may be prevented by rehydration and alkalinisation. Tetanus prophylaxis should be routine, and antibiotics may be required if signs of local infection develop. The bitten limb may require surgical debridement of necrotic tissue, but fasciotomy is rarely justified.

## Developing New Treatments to Reduce Local Tissue Damage

Hydrolases such as metalloproteinases, phospholipases A _2_, and possibly hyaluronidases are mostly responsible for the local tissue damage frequently observed in snake bite envenoming. It is very difficult for IgG and its fragments to neutralise these toxins, so there is an urgent need to develop new therapies to counteract local venom effects. Venom hydrolases are homologous with endogenous mammalian enzymes, such as matrix metalloproteinases, a disintegrin and metalloproteinase (ADAM) enzymes, and inflammatory phospholipases A _2_. The pharmaceutical industry has developed a large number of inhibitors to these mammalian enzymes because of their relevance in many pathologies. Since many of these drugs have already been tested in clinical trials, there is an excellent opportunity to use some of these inhibitors to prevent local effects of snake bite envenoming. For instance, the peptidomimetic hydroxamate metalloproteinase inhibitor “batimastat” is highly effective in preventing local tissue damage and some systemic effects induced by the venom of B. asper [ 18, 19]. Similarly, phospholipase A _2_ and hyaluronidase inhibitors have proven successful at reducing the extent of venom-induced tissue damage at the experimental level [ 20, 21]. The advantages of this strategy are the following: (1) such inhibitors are usually low-molecular-mass components that can diffuse rapidly in the tissues, and (2) such inhibitors would be effective against a wide variety of venom metalloproteinases and phospholipases A _2_ present in the venoms of different genera and species, since most of them act at the active sites of these enzymes, which are highly conserved.

## Towards More Effective and Safer Antivenoms

Despite the success of antivenoms in the therapy of snake bite envenoming, a number of research groups are working to improve the quality of antivenoms using different approaches: (1) new immunisation strategies, such as DNA immunisation or the use of purified relevant toxins as antigens instead of the whole venom [ 22]; (2) improved methods of antibody purification, aiming to obtain more refined products with reduced protein content [ 23]; (3) improved viral safety of antivenoms, by introducing steps such as pasteurisation, caprylic acid stabilisation, and nanofiltration [ 24]; (4) the search for other animal species from which it is possible to derive antivenoms—such as camels, which have a particular type of IgG devoid of light chains, and hens, which have IgY in their egg yolk [ 25]; and (5) preparation of antivenoms that combine complete antibodies with recombinant “nanobodies”, which by having a very low molecular mass may reach tissue compartments more readily than whole IgG or conventional IgG fragments [ 26]. In addition, there is a clear need for the design and development of controlled, randomised clinical trials in many countries to assess the effectiveness and safety of the antivenoms being used or introduced in such regions.

## Conclusion: The Need for a Global Partnership

**Figure 3:**
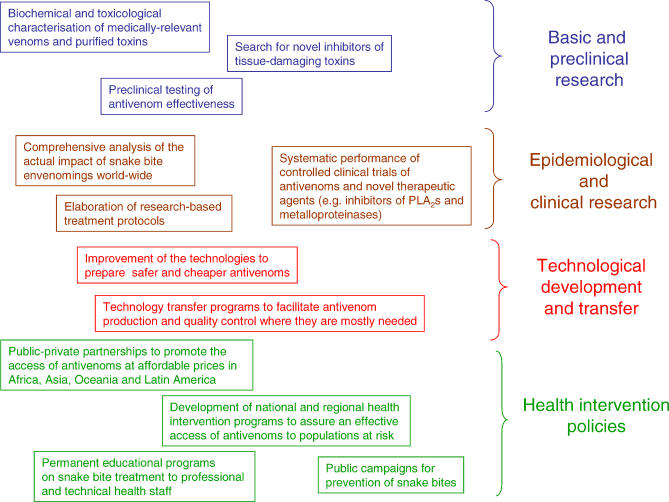
Scientific, Technological, and Political Tasks Required to Improve the Prevention and Treatment of Snake Bite Envenoming There is an urgent need to gain a deeper knowledge of the epidemiology and clinical manifestations of snake bite envenoming around the globe, and to promote research and public health policies aimed at improving the treatment and prevention of these envenomings, particularly regarding antivenom production and distribution, as well as training of medical staff. The fulfilment of these tasks requires a concerted effort involving many actors at both national and international levels. PLA _2_s, phospholipases A _2_.

In various parts of the world, there is a continuing crisis in the production, deployment, and accessibility of antivenoms. This is particularly serious in Africa since various traditional suppliers have stopped producing antivenoms for this continent, and the privatisation of other producers has also affected supply. It is therefore a matter of great urgency to promote international collaboration—best coordinated by the World Health Organization—involving national and regional health organisations and diverse public–private partnerships in order to accomplish the following goals: (1) to gather accurate epidemiological information on the impact and characteristics of snake bite envenoming in many regions, using properly designed community studies; and (2) to provide effective and safe antivenoms in those regions where they are not currently available. This includes, in the short term, the commitment of various laboratories to produce antivenoms for these regions, such as the efforts being undertaken by various producers to manufacture new antivenoms for use in Africa [ 27–29]. Innovative approaches to pharmaceutical laboratories, both public and private, should be initiated in order to foster improvement in antivenom supply [ 30, 31]. The long-term goal should be to generate an effective transfer of antivenom-producing technologies to regions hyperendemic for snake bites, to allow them to manufacture their own antivenoms ( Figure 3).

Besides the technological issues of antivenom production and supply, efforts should be devoted in Africa, Asia, Oceania, and Latin America to the preparation of consensus guidelines and protocols for treatment and prevention of snake bites [ 32], and to the establishment of teaching programmes in medical and nursing schools. There is a need to develop health intervention strategies, adapted for the particular needs of every country and region, to achieve more rapid access to antivenom in health facilities. Such integrated and global efforts, involving many participants, will undoubtedly reduce the global burden of human suffering inflicted by this neglected disease.

## Supporting Information

Alternative Language Abstract S1 Translation of the Article into Spanish by the Corresponding Author José Maria Gutiérrez (62 KB DOC).

Figure S1 Spanish Version of Figure 3, Translated by the Corresponding Author José María Gutiérrez
(39 KB PPT).
