# Supplementary material for: Confronting the Neglected Problem of Snake Bite Envenoming: The Need for a Global Partnership
Source: PLoS Med. 2006 Jun 6;3(6):e150. doi: 10.1371/journal.pmed.0030150 (PMC1472552; doi:10.1371/journal.pmed.0030150)
Supplement: Supplementary file 1 [file pmed.0030150.sd001.doc]

**NEGLECTED DISEASES**

Enfrentando el problema de los envenenamientos por mordedura de serpiente: necesidad de una alianza global

José María Gutiérrez*, R. David G. Theakston, David A. Warrell

José María Gutiérrez trabaja en el Instituto Clodomiro Picado, Facultad de Microbiología, Universidad de Costa Rica. R. David G. Theakston trabaja en la Liverpool School of Tropical Medicine, Liverpool, United Kingdom. David A. Warrell trabaja en el Nuffield Department of Clinical Medicine, University of Oxford, Oxford, United Kingdom

*Autor a quien se debe dirigir la correspondencia. Dirección electrónica: jgutierr@icp.ucr.ac.cr

Fuentes de financiamiento: J.M. Gutiérrez recibe apoyo de la Vicerrectoría de Investigación, Universidad de Costa Rica.

Conflictos de interés: Los autores declaran que no tienen conflictos de interés.

Derechos de autor: © 2006 Gutiérrez et al. Esta es una publicación de acceso libre distribuida bajo los términos del *Creative Commons Attribution License*, que permite el uso irrestricto, la distribución y la reproducción del material por cualquier medio, siempre y cuando se otorgue el crédito a los autores y se cite la fuente.

**Introducción**

Los envenenamientos por mordeduras de serpiente constituyen un problema de salud pública de gran importancia, especialmente en países tropicales y subtropicales [1-3]. Pese a que existen diversos laboratorios productores de antivenenos en todos los continentes, el impacto de estos envenenamientos, tanto en términos de morbilidad como de mortalidad, afecta enormemente a las poblaciones y a los sistemas sanitarios, especialmente en Africa, Asia, Oceanía y América Latina [4]. Desafortunadamente, las autoridades de salud pública, tanto a nivel nacional como internacional, le prestan muy poca atención a este problema, relegando así al envenenamiento por mordedura de serpiente a la categoría de una enfermedad desatendida en los albores del siglo XXI.

**El Impacto Global del Envenenamiento por Mordedura de Serpiente**

Existe muy escasa información confiable sobre la incidencia de los envenenamientos por mordedura de serpiente, especialmente en países en vías de desarrollo donde estos accidentes son más frecuentes. Uno de los esfuerzos más serios para cuantificar la mortalidad causada por estos envenenamientos fue el trabajo de Swaroop y Grabb, en 1954, el cual se basó fundamentalmente en datos de admisiones hospitalarias [3]. Estudios efectuados posteriormente revelaron un enorme subregistro de muertes en este informe en países como Nigeria [5] y Tailandia [1, 6]. Una razón para explicar esta situación es que los pacientes que son tratados por métodos empíricos tradicionales no entran en dichas estadísticas oficiales, y las muertes reportadas a nivel de distrito o comunidad frecuentemente no llegan a las bases de datos de los ministerios. No obstante, y aceptando estas limitaciones, la información disponible permite sugerir que, anualmente, ocurren varios millones de envenenamientos por mordedura de serpiente en el mundo, con decenas de miles de muertes [2, 3].

Los estudios poblacionales bien diseñados brindan un panorama mucho más preciso de la incidencia, morbilidad y mortalidad de estos envenenamientos. En Asia la mayor incidencia descrita fue de 162 muertes por mordedura de serpiente por 100.000 habitantes al año, en la región oriental de Terai, Nepal [7]. En el caso de Africa, se describió una incidencia de mordeduras de serpiente de 497 por 100.000 habitantes al año, con una letalidad del 12.2%, en el valle Benue, ubicado en la región noreste de Nigeria [5]. Las causas de esta elevada mortalidad en los países tropicales en vías de desarrollo incluyen la escasez de antivenenos, los deficientes servicios de salud y las enormes dificultades para trasladar a los pacientes a centros de atención médica [8]. Como consecuencia, muchos pacientes mueren o sufren secuelas físicas y psicológicas permanentes, debido sobretodo a los daños titulares inducidos por los venenos. El grupo más afectado corresponde a los trabajadores agrícolas jóvenes, principalmente varones, lo que convierte este problema en una típica enfermedad laboral, algo que es frecuentemente obviado por las autoridades nacionales. Además, los niños y las niñas son víctimas comunes de estos envenenamientos. Ocurre una importante variación estacional en la incidencia de estas mordeduras, la cual se asocia con aspectos climáticos que determinan los ciclos anuales de la actividad agrícola.

**Especies Responsables de la Mayor Morbilidad y Mortalidad**

Los casos más severos de envenenamientos por mordeduras de serpientes son causados por especies de las familias Elapidae (cobras, ‘kraits’, mambas, especies australianas y serpientes marinas) y Viperidae (cascabeles, otras víboras de foseta y víboras verdaderas). Las especies que causan el mayor número de mordeduras y muertes pertenecen a especies del género *Echis* sp en el norte de Africa, *Bothrops* *asper* y *B. atrox* en Centroamérica y Sudamérica, respectivamente, y las cobras (género *Naja*) y las ‘kraits’ (género *Bungarus*) en Asia [6,9-11] (Fig 1). Existen muchas otras especies que, aunque no representan un gran problema a escala global, sí causan muchas mordeduras en regiones específicas.

**Manifestaciones Clínicas del Envenenamiento por Mordedura de Serpiente**

Los envenenamientos causados por la mayoría de las serpientes de la familia Viperidae inducen un fuerte daño tisular local, caracterizado por edema, bulas, hemorragia y necrosis del tejido muscular (Fig 2). Las mordeduras de algunos elápidos, tales como ciertas cobras de Africa y Asia, también inducen necrosis local. Estos efectos patológicos locales son causados, principalmente, por fosfolipasas A_2_ y metaloproteinasas dependientes de zinc [12, 13]. Estos efectos locales tienen la particularidad de que se desarrollan muy rápidamente después de la mordedura, por lo que un retardo en el acceso al centro de salud trae como consecuencia un daño tisular importante y la aparición de secuelas permanentes. Las manifestaciones sistémicas también ocurren con frecuencia en estos envenenamientos y son causantes de letalidad. Las manifestaciones neurotóxicas son frecuentes en envenenamientos por elápidos, causadas por neurotoxinas con acciones pre- y/o post-sináptica a nivel de las uniones neuromusculares. Dichas toxinas causan una parálisis progresiva que puede ser letal cuando se afectan los músculos bulbares y respiratorios. En envenenamientos por elápidos, y por algunos vipéridos como las cascabeles sudamericanas, hay un cuadro de rabdomiólisis generalizada inducido por fosfolipasas A_2_, el cual se asocia con mioglobinemia, hiperkalemia e insuficiencia renal aguda. Las manifestaciones sistémicas en envenenamientos por vipéridos incluyen hemorragia espontánea, siendo la hemorragia cerebral la manifestación más seria, desfibrinogenación, coagulación intravascular diseminada y choque cardiovascular secundario a hipovolemia, vasodilatación y efectos directos sobre el miocardio. La insuficiencia renal aguda y el síndrome de distrés respiratorio agudo también son consecuencias frecuentes de estos envenenamientos. Existen muchas excepciones a este perfil fisiopatológico general descrito, dado que los proteomas de los venenos de serpientes incluyen toxinas con muy diversos efectos fisiopatológicos (para un panorama general de las manifestaciones clínicas de estos envenenamientos, véase la referencia [14]).

**Terapia del Envenenamiento por Mordedura de Serpiente: Antivenenos y Terapias Complementarias**

La administración intravenosa de antivenenos de origen animal (principalmente de caballos y ovejas) constituye la única terapia específica y eficaz para el envenenamiento por mordedura de serpiente. Existen muchos laboratorios productores de antivenenos, los cuales utilizan diversas metodologías para manufacturar tres tipos principales de productos, dependiendo de la naturaleza del principio activo. Algunos producen antivenenos constituidos por moléculas de IgG completas, pero la mayoría de los productores fabrican antivenenos constituidos por fragmentos F(ab’)_2_, obtenidos mediante digestión de la molécula de IgG con pepsina, en tanto unos pocos fabricantes producen antivenenos constituidos por fragmentos Fab, obtenidos por digestión de IgG con papaína [4, 15]. Las diferencias en la masa molecular de estos principios activos, así como otras características inmunológicas de los mismos, originan patrones farmacocinéticos disímiles, lo cual acarrea implicaciones farmacodinámicas, como por ejemplo el hecho de que con antivenenos Fab se ha descrito un fenómeno de recurrencia frecuente del envenenamiento [16]. La enorme variabilidad inmunoquímica de los venenos es la causa de que los antivenenos tengan una eficacia restringida a un espectro biológico y geográfico determinado.

Las investigaciones clínicas han demostrado que los antivenenos poseen una alta eficacia en la neutralización de las toxinas responsables de los efectos sistémicos, tales como hemorragia, coagulopatía y alteraciones hemodinámicas. También se ha descrito una reversión rápida de la neurotoxicidad causada por neurotoxinas de acción post-sináptica en envenenamientos por elápidos [1]. Por el contrario, los antivenenos no son eficaces en la reversión de la neurotoxicidad inducida por fosfolipasas A_2_ de acción pre-sináptica. La principal deficiencia de los antivenenos en términos de eficacia terapéutica se relaciona con la neutralización de los efectos patológicos locales [1]. Esta deficiencia no se debe a la ausencia de anticuerpos neutralizantes en los antivenenos, sino al desarrollo rápido de la patología local, que imposibilita el acceso de los anticuerpos neutralizantes al tejido antes de que ocurra el daño tisular [17].

El tema de la seguridad en la administración de los antivenenos es también importante dado que, si bien el perfil de seguridad de muchos antivenenos es satisfactorio, existen otros cuya administración se asocia con una alta incidencia de reacciones adversas tempranas y tardías [4, 15]. Los mecanismos precisos que median estas reacciones no se han identificado con certeza, aunque hay evidencias que apuntan a que las mismas se deben a activación directa del complemento, a los efectos de pirógenos contaminantes y a reacciones a complejos inmunes. Las características fisico-químicas de los antivenenos, principalmente la presencia de agregados de proteínas, la pureza del principio activo y la cantidad total de proteína administrada, parecen ser algunos de los principales factores determinantes de las reacciones adversas a los antivenenos. Las reacciones adversas tempranas son tratadas mediante la administración de adrenalina, anti-histamínicos y corticosteroides [6, 9].

La terapia del envenenamiento por mordedura de serpiente incluye, además del antiveneno, una serie de intervenciones complementarias [6]. La ventilación asistida es fundamental cuando los músculos respiratorios están comprometidos; así mismo, la administración de inhibidores de acetilcolinesterasa ha sido exitosa en algunos tipos de envenenamientos neurotóxicos [6]. La hipovolemia en envenenamientos por vipéridos se corrige mediante la administración de expansores del plasma y la insuficiencia renal aguda es tratada mediante diálisis y hemoperfusión. Las lesiones renales causadas por la acción de la mioglobina o la hemoglobina liberadas por rabdomiólisis o hemólisis intravascular pueden prevenirse con el uso de rehidratación y alcalinización. Se debe administrar profilaxis del tétano rutinariamente y la antibioticoterapia debe ser considerada si aparecen signos de infección local. La extremidad envenenada puede requerir desbridación de tejido necrótico y la fasciotomía se justifica en muy pocos casos.

**Desarrollo de Nuevos Tratamientos para Reducir el Daño Tisular Local**

Las hidrolasas de los venenos, principalmente las metaloproteinasas, las fosfolipasas A_2_ y posiblemente las hialuronidasas, son las responsables principales del daño tisular local observado en envenenamiento por mordedura de serpiente. La neutralización de estas toxinas por los anticuerpos de los antivenenos es muy pobre, por lo que se hace necesario desarrollar nuevas terapias para controlar los efectos locales del envenenamiento. Las hidrolasas de los venenos son homólogas a enzimas endógenas de mamíferos, tales como metaloproteinasas MMPs y ADAMs, así como fosfolipasas A_2_ inflamatorias. Debido a la relevancia que tienen estas enzimas de mamíferos en muchas patologías, la industria farmacéutica ha desarrollado una gran cantidad de inhibidores de estas enzimas. Dado que muchas de estas drogas han sido utilizadas en ensayos clínicos, existe una excelente oportunidad para utilizar este tipo de inhibidores en la prevención del daño tisular local en el envenenamiento por mordedura de serpiente. Por ejemplo, el inhibidor de metaloproteinasas ‘batimastat’, un hidroxamato peptidomimético, es muy efectivo en la inhibición del daño tisular local y de algunos efectos sistémicos inducidos por el veneno de *Bothrops asper* [18, 19]. De igual manera, inhibidores de fosfolipasas A_2_ y de hialuronidasas han sido efectivos en la reducción del daño tisular local inducido por ciertos venenos a nivel experimental [20, 21]. Esta estrategia de utilizar inhibidores en el envenenamiento por serpientes tiene varias ventajas: (a) dichos inhibidores poseen una masa molecular pequeña, lo que les permite una rápida difusión en los tejidos, y (b) estos inhibidores serían efectivos contra una gran cantidad de fosfolipasas A_2_ y metaloproteinasas de muy diversos venenos ya que la mayoría de los inhibidores actúan en el sitio activo de estas enzimas, el cual es generalmente muy conservado.

**Hacia el Desarrollo de Antivenenos Más Eficaces y Seguros**

Pese al indudable éxito que tienen los antivenenos en la terapia del envenenamiento por mordedura de serpiente, diversos grupos trabajan en varios frentes en el mejoramiento de la calidad de estos inmunobiológicos; algunos de estos esfuerzos son: (a) Nuevas estrategias de inmunización, tales como inmunización con ADN o el uso de toxinas purificadas como antígenos, en lugar de venenos completos [22]; (b) mejoramiento de los métodos de purificación de anticuerpos, con el objetivo de obtener productos más refinados con un contenido proteico menor [23]; (c) mejoramiento de la seguridad viral de los antivenenos, mediante la introducción de pasos como pasteurización, estabilización con ácido caprílico y nanofiltración [24]; (d) búsqueda de otros animales para producción de antivenenos, tales como los camellos, que presentan un tipo peculiar de IgG carente de cadenas livianas, y las gallinas, mediante la obtención de IgY a partir de la yema de huevo [25]; y (e) desarrollo de antivenenos que combinen anticuerpos completos con fragmentos de anticuerpos recombinantes los cuales, por su reducida masa molecular, puedan difundir a los tejidos más efectivamente que la IgG completa o los fragmentos F(ab’)_2_ [26]. Por otra parte, existe una gran necesidad de fortalecer el diseño y ejecución de ensayos clínicos aleatorizados en muchos países para evaluar rigurosamente la eficacia y seguridad de los antivenenos que estén siendo utilizados, o que vayan a ser introducidos, en dichas regiones.

**Conclusión: La Necesidad de Formar una Alianza Global**

Existe en la actualidad una crisis grave en la producción, distribución y accesibilidad de antivenenos en diversas regiones del mundo. Esta situación es particularmente seria en Africa, dado que los productores que tradicionalmente han fabricado antivenenos para dicho continente han suspendido en gran medida su producción, en tanto que la privatización de otros laboratorios también ha contribuido a esta crisis. Esta grave situación señala la urgencia de desarrollar renovados esfuerzos de cooperación internacional, coordinados por la OMS y con la participación activa de las instituciones de salud nacionales y regionales y de diversas alianzas público-privadas, con el fin de cumplir los siguientes objetivos: (a) Obtener información epidemiológica rigurosa, en gran escala, sobre el impacto y las características del envenenamiento por mordedura de serpiente en muchas regiones del mundo; y (b) garantizar el suministro de antivenenos seguros y eficaces en aquellas regiones donde se cuenta con este suministro actualmente. Esto incluiría, en el corto plazo, el compromiso de diversos laboratorios de producir antivenenos para dichas regiones, tal como el esfuerzo que se está efectuando para producir antivenenos para Africa [27-29]. Se deben proponer iniciativas novedosas a los laboratorios farmacéuticos, tanto públicos como privados, para incrementar el suministro de antivenenos [30, 31]. El objetivo a largo plazo, sin embargo, debe ser la transferencia de tecnologías de producción de antivenenos a regiones con alta incidencia en mordeduras por serpientes, con el fin de que dichas regiones produzcan sus propios antivenenos (Fig 3).

Además de los aspectos tecnológicos involucrados en la producción y suministro de antivenenos, se requiere trabajar en Africa, Asia, Oceanía y América Latina en la preparación de guías y protocolos de tratamiento y prevención de las mordeduras por serpientes [32] y en el establecimiento de programas de enseñanza de este tema en las facultades de Medicina y Enfermería. Existe una enorme necesidad, además, de desarrollar estrategias de atención en salud, adaptadas a las particularidades y requerimientos de cada país y región, que permitan garantizar un traslado más rápido de los pacientes envenenados a los centros de salud. Toda esta gama de esfuerzos, de carácter integrado y global, y en los que deben involucrarse muy diversos participantes, indudablemente contribuirá a reducir el impacto global de sufrimiento humano causado por esta importante enfermedad.

**Referencias**

1. Warrell DA (1992) The global problem of snake bite: its prevention and treatment. In: Gopalakrishnakone P, Tan CK, editors. Recent advances in toxinology research. Singapore: National University of Singapore.
2. Chippaux JP (1998) Snake-bites: appraisal of the global situation. Bull WHO 76: 515-524.
3. Swaroop S, Grab B (1954) Snakebite mortality in the world. Bull WHO 10: 35-76.
4. Theakston RDG, Warrell DA, Griffiths E (2003) Report of a WHO workshop on the standardization and control of antivenoms. Toxicon 41: 541-557.
5. Warrell DA, Arnett C (1976) The importance of bites by the saw-scaled or carpet viper (*Echis carinatus*). Epidemiological studies in Nigeria and a review of the world literature. Acta Trop (Basel) 33: 307-341.
6. Warrell DA (1995) Clinical toxicology of snakebite in Asia. In: Meier J, White J, editors. Handbook of clinical toxicology of animal venoms and poisons. Florida: CRC Press.
7. Sharma SK, Chappuis F, Jha N, Bovier PA, Loutan L, Koirala S (2004) Impact of snake bites and determinants of fatal outcomes in southeastern Nepal. Am J Trop Med Hyg 71: 234-238.
8. Theakston RDG, Warrell DA (2000) Crisis in snake antivenom supply for Africa. Lancet 356: 2104.
9. Warrell DA (1995) Clinical toxicology of snakebite in Africa and the Middle East/Arabian peninsula. In: Meier J, White J, editors. Handbook of clinical toxicology of animal venoms and poisons. Florida: CRC Press.
10. Gutiérrez JM (1995) Clinical toxicology of snakebite in Central America. In: Meier J, White J, editors. Handbook of clinical toxicology of animal venoms and poisons. Florida: CRC Press.
11. Fan HW, Cardoso JL (1995) Clinical toxicology of snake bites in South America. In: Meier J, White J, editors. Handbook of clinical toxicology of animal venoms and poisons. Florida: CRC Press.
12. Gutiérrez JM, Rucavado A (2000) Snake venom metalloproteinases: their role in the pathogenesis of local tissue damage. Biochimie 82: 841-850.
13. Gutiérrez JM, Ownby CL (2003) Skeletal muscle degeneration induced by venom phospholipases A_2_: insights into the mechanisms of local and systemic myotoxicity. Toxicon 42: 915-931.
14. Warrell DA (1996) Clinical features of envenoming from snake bites. In: Bon C, Goyffon M, editors. Envenomings and their treatments. Lyon: Fondation Marcel Mérieux.
15. Lalloo D, Theakston RDG (2003) Snake antivenoms. J Toxicol Clin Toxicol 41: 277-290.
16. Gutiérrez JM, León G, Lomonte B (2003) Pharmacokinetic-pharmacodynamic relationships of immunoglobulin therapy for envenomation. Clin Pharmacokinet 42: 721-741.
17. Gutiérrez JM, León G, Rojas G, Lomonte B, Rucavado A, Chaves F (1998) Neutralization of local tissue damage induced by *Bothrops asper* (terciopelo) snake venom. Toxicon 36: 1529-1539.
18. Rucavado A, Escalante T, Franceschi A, Chaves F, León G, Cury Y, Ovadia M, Gutiérrez JM (2000) Inhibition of local hemorrhage and dermonecrosis induced by *Bothrops asper* snake venom: effectiveness of early in situ administration of the peptidomimetic metalloproteinase inhibitor batimastat and the chelating agent CaNa_2_EDTA. Am J Trop Med Hyg 63: 313-319.
19. Rucavado A, Escalante T, Gutiérrez JM (2004) Effect of the metalloproteinase inhibitor batimastat in the systemic toxicity induced by *Bothrops asper* snake venom: understanding the role of metalloproteinases in envenomation. Toxicon 43: 417-424.
20. Angulo Y, Lomonte B (2003) Inhibitory effects of fucoidan on the activities of crotaline snake venom myotoxic phospholipases A_2_. Biochem Pharmacol 66: 1993-2000.
21. Yingprasertchai S, Bunyasrisawat S, Ratanabanangkoon K (2003) Hyaluronidase inhibitors (sodium chromoglycate and sodium auro-thiomalate) reduce the local tissue damage and prolong the survival time of mice injected with *Naja kaouthia* and *Calloselasma rhodostoma* venoms. Toxicon 42: 635-646.
22. Harrison RA, Moura-da-Silva AM, Laing GD, Wu Y, Richards A, Broadhead A, Bianco AE, Theakston RDG (2000) Antibody from mice immunized with DNA encoding the carboxyl-disintegrin and cysteine-rich domain (JD9) of the hemorrhagic metalloproteinase, jararhagin, inhibits the main lethal component of viper venom. Clin Exp Immunol 121: 358-363.
23. Grandgeorge M, Véron JL, Lutsch C, Makula MF, Riffard P, Pépin S, Scherrman JM (1996) Preparation of improved F(ab^/^)_2_ antivenoms. An example: new polyvalent European viper antivenom (equine). In: Bon C, Goyffon M, editors. Envenomings and their treatments. Lyon: Fondation Marcel Mérieux.
24. Burnouf T, Griffiths E, Padilla A, Seddik S, Stephano MA, Gutiérrez JM (2004) Assessment of the viral safety of antivenoms fractionated from equine plasma. Biologicals 32: 115-128.
25. Thalley BS, Carroll SB (1990) Rattlesnake and scorpion antivenoms from the egg yolks of immunized hens. Biotechnology 8: 934-938.
26. Harrison RA, Hasson SS, Harmsen M, Laing GD, Conrath K, Theakston RDG (2005). Neutralisation of venom-induced haemorrhage by IgG from camels and llamas immunized with viper venom and also by endogenous, non-IgG components in camelid sera. Toxicon (in press).
27. Laing GD, Renjifo JM, Ruiz F, Harrison RA, Nasidi A, Gutiérrez JM, Rowley PD, Warrell DA, Theakston DA (2003) A new Pan African polyspecific antivenom developed in response to the antivenom crisis in Africa. Toxicon 42: 35-41.
28. Gutiérrez JM, Rojas E, Quesada L, León G, Núñez J, Laing GD, Sasa M, Renjifo JM, Nasidi A, Warrell DA, Theakston RDG, Rojas G (2005) Pan-African polyspecific antivenom produced by capryic acid purification of horse IgG: an alternative to the antivenom crisis in Africa. Tr R Soc Trop Med Hyg 99: 468-475.
29. Chippaux, JP, Stock RP, Alagón A (2005) Report of the 2^nd^ International Conference of Envenomations in Africa (Deuxieme Colloque International sur les Envenimations en Afrique). Toxicon 46: 115-118.
30. Cheng AC, Winkel K (2001) Call for global snake-bite control and procurement funding. Lancet 357: 1132.
31. Wilde H, Thipkong P, Sitprija V, Chaiyabutr N (1996) Heterologous antisera and antivenins are essential biologicals: perspectives on a worldwide crisis. Ann Intern Med 125: 233-236.
32. Warrell DA (1999) WHO/SEARO guidelines for the clinical management of snake bites in the Southeast Asian region. Southeast Asian J Trop Med Public Health 30 (Supplement 1): 1-85.

**Leyendas de las Figuras**

**Figura 1**. (A) *Echis ocellatus* (familia Viperidae, de Nigeria), (B) *Naja naja* (‘cobra’, familia Elapidae, de Sri Lanka) y (C) *Bothrops asper* (‘terciopelo’ o ‘equis’, familia Viperidae, de Costa Rica) son responsables de muchos envenenamientos en Africa sub-Sahariana, en Asia y en Centroamérica, respectivamente. Los envenenamientos por *E. ocellatus* y *B. asper*, así como por otras serpientes vipéridas, inducen daño tisular local severo, el cual con frecuencia ocasiona secuelas permanentes, y alteraciones sistémicas asociadas a hemorragia, coagulopatía, choque cardiovascular e insuficiencia renal. Los envenenamientos por *N. naja* y otras cobras asiáticas inducen efectos neurotóxicos sistémicos y necrosis local. Fotografías (A) y (B) por D.A. Warrell; fotografía (C) por Mahmood Sasa.

Figura 2. Necrosis tisular masiva en la extremidad inferior en un niño de 11 años, dos semanas después de una mordedura por *Bothrops atrox* en Ecuador. Únicamente se le suministró terapia de antibióticos, por lo que el daño tisular fue masivo, requiriendo amputación. Este tipo de alteraciones patológicas son causadas por la acción directa de toxinas de acción local (principalmente metaloproteinasas y fosfolipasas A_2_), conjuntamente con los efectos indirectos generados por los eventos inflamatorios, así como por la isquemia resultante del daño vascular inducido por el veneno y el incremento en la presión intracompartimental. Fotografía por D.A. Warrell.

**Figura 3**. Tareas científicas, tecnológicas y políticas requeridas para mejorar la prevención y el tratamiento del envenenamiento por mordedura de serpiente. Existe una necesidad urgente de profundizar en el conocimiento de la epidemiología y las manifestaciones clínicas de estos envenenamientos a escala global. También se requiere promover la investigación y la implementación de políticas de atención en salud dirigidas al mejoramiento en el tratamiento y la prevención de estos envenenamientos, especialmente en lo que se refiere a producción y distribución de antivenenos, así como a capacitación del personal de salud que atiende estos casos. El cumplimiento de estas tareas demanda esfuerzos compartidos de muchos actores a escalas nacional e internacional.
